# Directly Predicting Water Quality Criteria from Physicochemical Properties of Transition Metals

**DOI:** 10.1038/srep22515

**Published:** 2016-03-03

**Authors:** Ying Wang, Fengchang Wu, Yunsong Mu, Eddy Y. Zeng, Wei Meng, Xiaoli Zhao, John P. Giesy, Chenglian Feng, Peifang Wang, Haiqing Liao, Cheng Chen

**Affiliations:** 1 State Key Laboratory of Environmental Criteria and Risk Assessment, Chinese Research Academy of Environmental Sciences, Beijing 100012, China; 2 College of Water Sciences, Beijing Normal University, Beijing 100875, China; 3 School of Environment, Guangzhou Key Laboratory of Environmental Exposure and Health, and Guangdong Key Laboratory of Environmental Pollution and Health, Jinan University, Guangzhou 510632, China; 4 Department of Veterinary Biomedical Sciences, and Toxicology Centre, University of Saskatchewan, Saskatoon, SK, Canada; 5 Department of Zoology, and Center for Integrative Toxicology, Michigan State University, East Lansing, MI, USA; 6 College of Environment, Hohai University, Nanjing 210098, China

## Abstract

Transition metals are a group of elements widespread in aquatic environments that can be hazardous when concentrations exceeding threshold values. Due to insufficient data, criteria maximum concentrations (CMCs) of only seven transition metals for protecting aquatic life have been recommended by the USEPA. Hence, it is deemed necessary to develop empirical models for predicting the threshold values of water quality criteria (WQC) for other transition metals for which insufficient information on toxic potency is available. The present study established quantitative relationships between recommended CMCs and physicochemical parameters of seven transition metals, then used the developed relationships to predict CMCs for other transition metals. Seven of 26 physicochemical parameters examined were significantly correlated with the recommended CMCs. Based on this, five of the seven parameters were selected to construct a linear free energy model for predicting CMCs. The most relevant parameters were identified through principle component analysis, and the one with the best correlation with the recommended CMCs was a combination of covalent radius, ionic radius and electron density. Predicted values were largely consistent with their toxic potency values. The present study provides an alternative approach to develop screening threshold level for metals which have insufficient information to use traditional methods.

Transition metals are a group of elements in groups IIIB to IIB of the Periodic Table of the elements. The last electron in a transition metal normally fills the secondary outer layer *d* orbital, resulting in low ionization energies and various, multiple valences. Transition metals, with richer chemical characteristics than main-group elements^1^, are an important class in the Periodic Table. They are widespread in aquatic environments, mostly at low concentrations, but can exert detrimental effects on aquatic life and human health. Water quality criteria (WQC) are the scientific foundation for assessment or qualities of aquatic environments and risk management. The United States Environmental Protection Agency (USEPA) published the first WQC guidelines, referred to as the “Red Book”, in 1976. The document proposed criteria maximum concentrations (CMCs) for nine transition metals^2^. The USEPA has subsequently updated WQC guidelines seven times in the past 40 years^3^,^4^,^5^,^6^,^7^,^8^,^9^. In the latest guideline, the USEPA recommended CMCs for only 10 metals for protecting aquatic life; seven of them are transition metals^8^, i.e., chromium (Cr (III), Cr (VI)), nickel (Ni), copper (Cu), zinc (Zn), silver (Ag), cadmium (Cd) and mercury (Hg).

Due to the lack of data on toxic potency of metals, WQC for more than 50 other transition metals have not yet been promulgated by regulatory jurisdictions. The reason for this is a general lack of empirical information on toxic potencies of these elements to model aquatic species, which is needed to derive WQC^10^. However, because tests to determine toxic potencies are often costly and time-consuming, they are not available for many species, and in particular rare for endangered species that would be the focus of protective WQC. Furthermore, toxicities of some non-essential transition metals are greater than those of the main-group elements.

There are two indirect methods that have been used to predict toxic potency of metals for which toxicity data were insufficient. The first method is the interspecies correlation estimations (ICE) model, intended for species that can not be tested and is therefore used to extrapolate from toxicity data for surrogate species^11^. The second method is based on quantitative structure-activity relationships (QSARs), which are correlations between physicochemical properties and toxic potencies of target compounds^12^,^13^. These methods are not adequate for data-poor, non-essential transition metals for which data are available for only surrogate or common species. Therefore, new methods to directly predict WQC of transition metals using minimal toxic data were desirable.

Using fewer species and making better predictive models are the future integrated strategies of toxicology^14^. Critical mechanisms of toxicities of metals are often associated with their electronic structures and key physicochemical properties, such as binding affinity with biological macromolecular ligands^15^. Hence it has been proposed that physiochemical parameters can be used to develop models to predict toxic potencies of metals^16^. Because they are similar in electronic structures, transition metals can have similar physicochemical properties and mechanisms of toxicity^17^. For example, more than 20 physicochemical parameters, including softness, hydrolyzability, ionizability, complexing ability and geometric characteristics, have been shown to correlate with biological activities^16^. Alternatively, methods recommended by the USEPA, such as toxicity centile rank, SSDs and evaluation factors, all utilize data on toxic potency to several species to derive both WQC and CMCs^18^.

To demonstrate this structural property-based approach, empirical relationships between the USEPA-recommended CMCs and physicochemical properties of seven transition metals were established. After the most relevant parameters were selected, a model was established to predict CMCs of 49 other transition metals in the fourth, fifth, sixth and seventh periods of the Periodic Table of the elements, including the Lanthanide and Actinide Series. The predicted values were then compared with toxicity data from the literature, so as to examine the utility and reliability of the predictive model.

## Results and Discussion

### Single Physicochemical Properties-CMCs Relationships of Transition Metals

Twenty-six descriptors of physicochemical properties were considered in constructing models to predict CMCs by use of single-parameter linear regressions (Table 1). Seven structural parameters, including atomic number (*AN*), relative atomic weight (*AW*), covalent radius (*CR*), Pauling ionic radius (*r*), atomic ionization potential (*AN/∆IP*), softness index (*σp*) and electron density (*AR/AW*), were found to reasonably correlate with the CMCs of the seven transition metals recommended by the USEPA (*R*^*2*^ > 0.5 and *P* < 0.05; Table 2). It is therefore possible to develop empirical models by use of physicochemical properties and recommended CMCs for the seven transition metals, which can be employed to predict CMCs of other transition metals.

The parameters, *AN, AW, CR, r* and *AN/∆IP* were significantly and negatively correlated with CMCs (Supplementary Fig. S1A–E). This result is consistent with previously reported findings that the toxic potency of a metal is determined by its electronic configuration (*AN/∆IP*)^16^, *AN*^16^,^19^ and *AW*^19^. Significant correlations between LD_50_ and *AN* for some mammalian and between EC_50_ and *AW* of *Daphnia magna* have also been reported^20^,^21^. *AN/∆IP*, represents the difficulty of metal ions to form covalent bonds due to configurations of their electrons and subsequent crystalline structures. In addition, *∆IP* is an indicator of change in ionization potential between ion oxidation numbers *OX* and *OX*^−1^. As a result, the potential for forming stable complexes between metal ions and biological ligands is directly related to toxic potencies of transition metals. Previous studies also indicated that *AN/∆IP* was negatively correlated with log EC_50_ (median effect concentration) of *Lymnaea acuminata* and LC_50_ (median lethal concentration) of *Caenorhabditis elegans*^22^,^23^. Parameters *CR* and *r* comprehensively describe the propensity of metal ions to form covalent and ionic bonds. In a similar study, Enache *et al*.^24^ noticed that increased inherent toxicity of metals was generally accompanied with increasing *AN, CR* and *r* of cabbage plants (*Brassica oleracea* L var *capitata* cv Soshu).

Alternatively, *σp* and *AR/AW* are positively correlated with CMCs, such that ions of metals with stronger hydrolysis and ionization potential have lesser toxic potency to aquatic organisms (Supplementary Fig. S1F,G). The softness index *σp*, derived by application of the Hard-Soft-Acid-Base (HSAB) theory, is indicative of the ability of metal ions to lose their valence electrons, while *AR/AW* is regarded as a measure of the electron density of ions. The results presented herein are consistent with those of previous studies. For instance, significant positive correlations between *σp* and LD_50_ determined in toxicity tests with mice were obtained for all hard, soft and borderline metal ions^25^. A positive correlation between *AR/AW* and EC_50_ values was also noted^21^.

Moreover, the two parameters with the largest coefficients of determination (*R*^*2*^) in PPCR models are *σp* (*R*^2^ = 0.75; *F* = 17.6 and *P* = 0.006) and *CR* (*R*^2^ = 0.62; *F* = 9.9 and *P* = 0.020). Consistently, *σp* is significantly and positively correlated with logEC_50_ and is the single best parameter used to predict relative toxic potencies of metal ions to a range of species, including *Vibrio fischeri, Helianthus annuus* Sunspot, and four arthropods (*Chironomus tentans, Planaria, Crangonyx pseudogracilis* and *Daphnia magna*)^22^,^23^,^26^. However, in contrast to the results obtained in the present study, Khangarot *et al*.^27^ observed no significant correlation between CR and EC_50_ of *Cypris subglobosa*. The reason for such a discrepancy may be that these authors investigated the sensitivities among metals for a single species, whereas we considered threshold values for protecting all aquatic organisms.

### Development of an Integrated Radius-PPCR Model

It has been difficult to predict relative potencies of metals by use of a single structural parameter^13^. Thus, it might be more appropriate to use common and easy-to-obtain physiochemical properties^28^. Because values for *σp* and *∆IP* were scarce, data for *AN, AW, CR, r* and *AR/AW*, which are readily available, were used to predict CMCs. However, because there were multiple parameters with correlation coefficients greater than 0.65, the information produced by the models described in the preceding section was somewhat redundant. To address this issue and extract canonical relationships, PCA was used to reduce the number of independent variables to a small set of integrated variables. Contributions to PC by the reduced number of variables were determined all autocorrelations eliminated.

Because coefficients of determination of pairwise correlations between *AN, AW* and *AW/AR* were all greater than 0.87, PCA analyses were conducted on four different combinations of the parameters: (1) *CR, r* and *AR/AW*; (2) *AN, CR* and *r*; (3) *AW, CR* and *r* and (4) all five parameters. The accumulated proportions of the first PC were 88.8%, 87.8%, 85.7% and 85.0%, respectively, for the four PCA analyses (Table 3). Thus, the first PCs were all selected to construct the PPCR models with single-parameter linear regression (Table 3). Among the four regressions, *X*_1_* *=* 0.567CR *+* 0.568r* − *0.597AR/AW* was the best fitted (*R*^2^ = 0.63, *F* = 10.2, *P* = 0.019). The results of internal cross-validation for the finally selected model was *Q*_*cv*_^*2*^ = 0.55 and *RMSE*_*cv*_ = 0.32, which demonstrated that the model was robust. In addition, the results of the applicability domains were acceptable, indicating that the model could be applied for predicting CMCs of other metal (Supplementary Fig. S2, S3). Herein, *X*_*1*_ is defined as integrated radius (IR) related to *AR, CR* and *r*, which are all basic parameters for describing metal properties including toxic potency^29^.

Some chemical and biological characteristics associated with adsorption and migration of ions are related to *r*^30^. For example, toxic potencies of metal ions are determined from their atomic orbital energies and *r*, and metal ions with greater toxic potency mostly have multiple oxidation states^31^. In general, *r* and *CR* can be calculated from nuclear charge and electron configuration^16^. *CR* is also related to *r*^32^. IR accounts for the effects of the radius on toxicity and also averts redundancy. Thus, it was more accurate than a single parameter for predicting CMCs.

**Figure 1:**
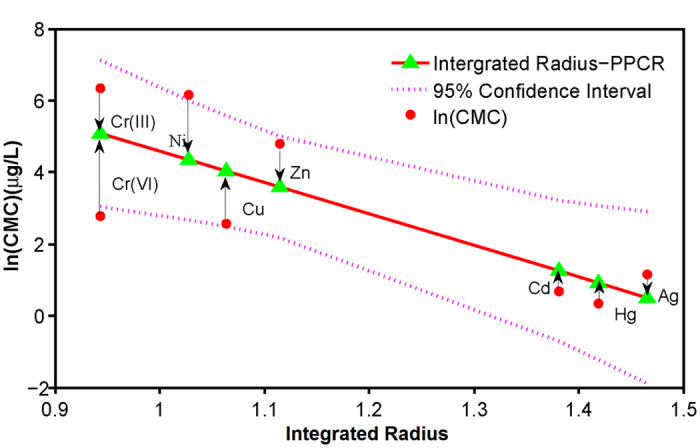
Predictive model for Criteria Maximum Concentrations (CMCs) on a natural logarithmic scale and integrated radius (*X*_*1*_) at 95% centile. Data points of CMCs predicted from integrated radius (IR) are plotted as 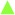, and the data points for USEPA-recommended CMCs are plotted as 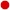. The purple, dashed line illustrated the 95% confidence interval.

The IR-PPCR model (Fig. 1) predicted CMCs for all seven metals except for that of Cr were within the 95% confidence intervals of the CMCs predicted from IR. In addition, the difference between the CMC for Hg predicted by IR and the recommended value was within ± 0.20, whereas differences for all other metals were within an order of magnitude. These results suggest that the model based on IR is capable of reliably predicting CMCs for transition metals. The WQC for Cu derived by the SSD approach was 30 ± 0.61^33^ and 48 ± 0.27 μg/L^34^, which is close to the predicted values of 39 and 35 μg/L obtained in the present study. As for Cr, the difference between the predicted and recommended values of CMCs is greater, probably because Cr has different valence states.

Three factors can explain uncertainties due to the use of different radii in IR, which was responsible for the discrepancy between predicted and recommended CMCs of the seven transition metals. First, substantially different predictions may be obtained if different ion radii are used. The radii reported by different groups for the same metal are not always identical^35^, and an ion radius can be classified as several types, including Lande, Wasastjerna, Goldschmidt or Pauling^36^. The inter-nuclear distance between a positive and a negative ion is the sum of their radii, but the boundary between them is quite difficult to determine. Second, both Cu and Zn always occur as +2 cations in freshwater, and thus can form stable complexes with hydroxo and carbonato- complexes^37^. The order of stability constants for +2 cations of first-row transition metals to form a complex with a ligand, called Irving-Williams stability series, is Cd^2+^ < Mn^2+^ < Fe^2+^ < Co^2+^ < Ni^2+^ < Cu^2+^ > Zn^2+^. Because Zn uses 4*s*4*p*^2^ tetrahedral orbitals, it often forms weaker complexes with organic ligands than other transition metals^38^. The effect of the ligand field in this case may cause uncertainties associated with *r* values used in the present study. Finally, *AR* can not be determined directly, and it is often measured with the assumption that the structure of metal atoms is spherically symmetrical. Similar to *r*, different values of the same metal radius also can be measured and calculated by different groups, such as Slater^36^ and Pauling^39^. Therefore, the use of different *AR* values may have caused the different results.

### Prediction and Comparison of Criteria Maximum Concentrations

**Figure 2:**
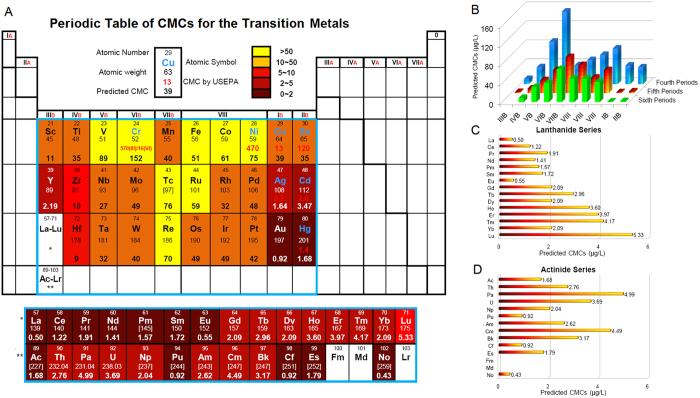
Predicted Criteria Maximum Concentrations (CMCs). (**A**) Periodic Table of CMCs for transition metals, showing CMCs recommended by US EPA and predicted by the integrated radius-PPCR (Physicochemical Properties-CMCs Relationships) model. (**B**) The predicted CMCs of the lanthanides. (**C**) The predicted CMCs of the actinides. (**D**) Comparison among the predicted CMCs in the forth (blue), fifth (red) and six period (green). The x axis of this graph is the group from IIIB to IIB, the y axis of the graph is the concentrations of the predicted CMCs, and the z axis is the periods.

CMCs of 56 transition metals in the fourth, fifth, sixth and seventh periods, including the lanthanide series and the actinide series, were predicted from the IR-PPCR model (Fig. 2A). Predicted CMCs of the lanthanides and actinides are similar (Fig. 2B,C). To facilitate pattern recognition, metals of the same period are divided into three groups, i.e., IIIB−VIIB, VIII and IB−IIB. Within the same period, CMCs increase with increasing atomic number for all three groups (Fig. 2D). Within the same group of the Period Table, CMCs are inversely proportional to atomic number (Fig. 2D).

**Figure 3:**
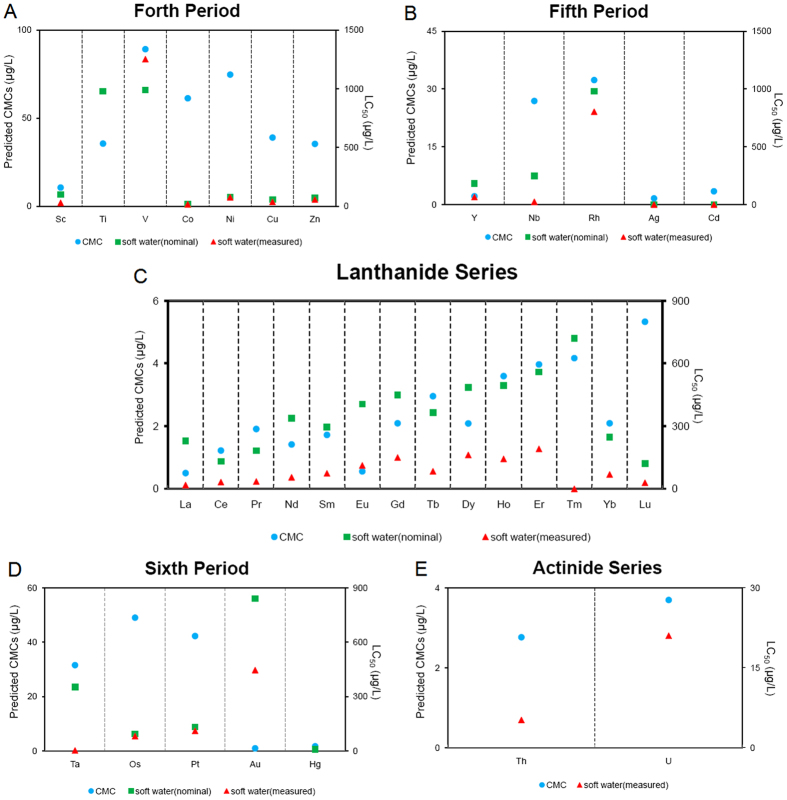
Comparison among Criteria Maximum Concentrations (CMCs) predicted by the model based on integrated radius (IR) (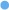), median lethal concentration (LC_50_) for the fresh water amphipod (*Hyalella azteca*, Crustacea) in Lake Ontario (Burlington city tap, Canada) in soft water (nominal) (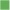) and soft water (measured) (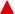), for seven transition metals in the fourth period (**A**), five transition metals in the fifth period (**B**), five transition metals in the sixth period (**D**), 14 lanthanide series metals (**C**) and two actinide series metals (**E**).

Median acute, lethal (LC_50_) concentrations, determined for 31 transition metals in one-week exposure experiments with *Hyalella azteca* (Crustacea) collected from Lake Ontario^40^, were correlated with the predicted CMCs. Exceptions were observed for yttrium (Y) and niobium (Nb), Cu and Zn, Ag and Cd, and gold (Au) and osmium (Os) (Fig. 3A,B,D). Within the same group, the sequences of LC_50_ concentrations and predicted CMCs are identical for the pairs of vanadium (V) and Nb, Cu and Ag, and Zn and Cd (Table 4). In addition, differences between LC_50_ concentrations or predicted CMCs with respect to atomic number are similar for the lanthanide and actinide series (Fig. 3C,E), probably because they are comparable in electronic structures in outside orbitals. If nominal LC_50_ concentrations^40^ are used for comparison, their sequences are the same as those of predicted CMCs for Y and Nb, Ag and Cd, and Nb and tantalum (Ta) (Table 4). It should be noted that Au was excluded from the above assessment because there are insufficient toxicity data for this relatively unreactive metal.

Toxic potency values expressed as CMCs and LC_50_ are similar between the lanthanide and actinide series metals (Fig. 3C,E). The lanthanides and actinides are similar in configurations of outside orbitals of electrons, which explains why most of their physical and chemical properties are similar. Lanthanides and actinides are also distinctly different from other elements in terms of physical and chemical properties because they have electrons in the *f* orbitals. The energy of the 4*f* sub-shell of lanthanides is lower than that of the 5*d* sub-shell for lanthanide metals, hence electrons fill the 4*f* sub-shell before the 5*d* sub-shell^41^. The “Lanthanide contraction”, another important feature of the lanthanide series in which the 5*s* and 5*p* orbitals penetrate the 4*f* sub-shell, results in the 4*f* orbital being exposed to the increasing nuclear change^42^. As a result, the atomic radius exhibits a decreasing trend throughout the series. This change in “charge density” might explain the difference in toxic potencies among the lanthanides. Therefore, the *r* and other physicochemical properties of the lanthanide metals beyond Eu in the Period Table are similar to those of Y, and these metals have similar LC_50_ concentrations and predicted CMCs as Y.

Actinides can form chemical compounds in solutions as cations with relatively large ionic radii^43^. Similar to the lanthanides, energies of the 6*s* and 6*p* sub-shells of actinides are greater than that of the 5*f* sub-shell; therefore electrons fill the 5*f* sub-shell before the 6*s* and 6*p* sub-shells. Therefore, both the lanthanides and actinides have the ability to form stable complexes with ligands, such as chloride, sulfate, carbonate and acetate. Moreover, some lanthanides and all actinides are radioactive^44^,^45^ and also exhibit characteristics of heavy metals, such that they are often considered toxic to aquatic life at ambient concentrations^46^. These results further corroborate the accuracy of the model based on IR in predicting toxic potency of metals (Fig. 2).

There is an apparent difference in the patterns of toxic potencies and predicted CMCs for some transition metals, probably since only the metal physiochemical properties are considered in the model based on IR, without considering effects of characteristics of natural water. To predict the effects in surface waters, the results predicted by the model need to be adjusted to account for metal speciation and chemical activity or apparent concentrations in both fresh and marine water. Due to cation competition and formation of biotic ligands by use of models that predict metal speciation by combining with the Biotic Ligand Model (BLM), free ion activity model (FIAM) and gill surface interaction model (GSIM)^47^. The BLM assesses metal toxicity to aquatic organisms over a range of hardness, pH and dissolved organic carbon (DOC) by providing a quantitative framework^48^ and has been employed as a good solution to the problems associated with WQC for Cu^49^. However, it has been only used to predict the toxicity of a few metals such as Cu, Ag, Cd and Ni to a few species, including *Salmo gairdneri, Pineohales pronelas, Daphnia magna, Ceriodaphnia dubia* and *Daphnia pulex*^50^. In general, if the BLM can not be used, the toxicity data used to derive WQC need to select under a constant pH such as ranged from 6 to 8 and be hardness-normalized by use of hardness algorithms, for which might be not concerned about effect of organic complexation. While further development and improvements of the predictive model are necessary and their range of applicability needs to be determined, the predictive model provides a promising screening level tool that can be used for rapid prediction of the criteria of the metals without any toxicity data and water quality and risk assessment.

### Importance and Uncertainties

Transition metals under investigation in the present study behave variably due to their individual physical and chemical properties; they have been widely used not only in industrial products but also in daily life. However, most transition metals exhibit significant toxic potency, some of them are even radioactive. Because of the difficulty to conduct experiments on these transition metals, there are few data on toxic potency to a range of species. As a result, it is difficult to establish water quality standards, conduct water quality assessment and practice risk management. Models obtained in the present study could be useful for deriving threshold values for data-poor transition metals. More importantly, the results of the present study demonstrated correlations between the physicochemical properties of transition metals and WQC and toxic potencies of metals. The modeling approaches used in the present study have also opened up a new dimension for investigating the complex environmental behavior and toxic action of transition metals, which is important for examining toxic potency and threshold values for other metals as well.

Although the IP-based model developed in the present study can reasonably predict CMCs of transition metals with limited information, experimental verification and subsequent modifications of the model are deemed necessary in future studies. In addition, the metal valence and the effects of water chemistry on toxic potency should also be considered in further modifications of the model. Nevertheless, the predictive model provides a new approach for WQC development and water quality assessment of metals.

## Methods

### Preparation of CMCs and Physicochemical Properties of Selected Transition Metals

Seven transition metals (Cr(III), Cr(VI), Ni, Cu, Zn, Ag, Cd and Hg) for which CMCs have been recommended by the USEPA^9^ were selected as “test elements” in the training set of elements, to which the results of the predictive models could be compared. Based on the results of several previous studies^12^,^28^,^51^,^52^,^53^,^54^, 26 structural parameters characterizing various physical and chemical properties of the metal ions were investigated. They include *AN*^12^, *AW*^28^,^52^, *AR*^28^,^52^,^53^,^55^, *CR*^51^,^52^,^53^, *r*^12^,^28^,^52^,^53^, melting point (*MP*)^52^, density (*D*)^52^, enthalpy (heat) of vaporization (*Eh*)^52^, boiling point (*BP*)^52^, difference in ionization potentials between the ion oxidation numbers *OX* and *OX*^−1^ (Δ*IP(eV)*)^12^,^28^,^54^, electrochemical potential (∆*E*_*0*_*(V)*)^12^,^28^,^52^,^54^, log of first hydrolysis constant (*|logK*_*OH*_*|*)^12^, covalent index (*X*_*m*_^*2*^*r*)^12^,^24^, polarization force parameters (*Z/r,Z/r*^*2*^ and *Z*^*2*^*/r*)^12^,^24^, *σp*^12^, ionization potential (*IP*)^12^,^23^,^24^,^26^, electronegativity (*X*_*m*_)^12^,^23^,^24^, *AN/∆IP*^12^,^23^,^24^,^26^, *AR/AW*^23^,^24^, electronegativity index (*x*)^12^,^23^, relative softness (*Z/rx*) (x is a electronegativity value index)^12^,^23^,^24^, similar polarization force parameters (*Z/AR* and *Z/AR*^2^)^12^,^28^,^52^,^53^ and ionic charge (*Z*)^26^. Some of these parameters such as *Z/AR,Z/rx* and *Z*^*2*^*/r* were recalculated to fit the model. Moreover, because the variables used to describe environmental concentrations often follow a lognormal frequency distribution, values of the descriptors were transformed to natural logarithm before use^56^.

### Statistical Analysis

Based on results of Pearson correlations analysis, 26 parameters were correlated with CMCs of the target metals recommended by the USEPA (Table 1), so that relationships between physicochemical properties and CMCs could be developed. Selected parameters and CMCs were used as independent and dependent variables, respectively. These Physicochemical Properties-CMCs Relationships (PPCR) models were developed based on multiple linear regressions of those parameters with the greatest correlations and thus predictive power. Selected parameters and CMCs were used as independent and dependent variables, respectively. Principal component analysis (PCA) was used to manage multivariate variables by transforming relationships from a higher-dimensional space to a lesser-order dimensional space, which simplified and optimized the information in the multivariate data. After linear regression of the original variables, several newly created variables expressed as principal components (PCs) can optimally represent the dynamic and interactive relationships among the original variables^57^. Since these comprehensive indices are perpendicular and minimally related, they can provide key non-redundant information about the original parameters. The first principal component (PC) generally explains the largest portion of the variation. While the number of PCs derived is equal to the total number of parameters included in the PCA, the number of PCs was chosen in the model so that greater than 85% of the total variance could be explained^58^. By using the PCA regression approach, the best correlation between the first principal component *X*_*1*_ and the recommended CMCs of the target metals was obtained. The model obtained by linear regression was used to predict CMCs for other transition metals. Principal component linear regression analyses were carried out by use of the R programming language and MATLAB (Mathworks, Natick, MA, USA). The predictive potential of the model was evaluated with the coefficient of determination (*R*^2^), residual standard error (RSE), the value of *F-*test statistic using analysis of linear regression fit and the level of Type I error (*P*) with the level of significance at α < 0.05.

### Model Validation

To reduce the probability of over-fitting and test the robustness of the model, internal validation was evaluated with k-fold cross-validation correlation coefficient (*Q*_*cv*_^2^), for which recommended minimum acceptable value is 0.5, and cross-validated root mean square error of prediction (*RMSE*_*cv*_)^59^. Moreover, predictions of WQC and toxic potencies of metals are valid only if the properties of such metals are within the applicability domains of the developed QSAR models. The applicability domains of the developed QSAR models were evaluated with the hat value and Williams plot^60^. The hat value h_i_ for each i^th^ metal was calculated with 
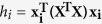
, where x_i_ is a row vector of the parameter for an i^th^ metal used to establish the QSAR model. The hat value h_i_ should be smaller than the warning h* value, i.e., the predicted CMC of an i^th^ metal is located within the optimum applicability domains. The h* value was calculated with 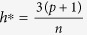, where *p* is the variables number used in the model, and *n* is the number of recommended CMCs for metals.

## Additional Information

**How to cite this article**: Wang, Y. *et al*. Directly Predicting Water Quality Criteria from Physicochemical Properties of Transition Metals. *Sci. Rep.* **6**, 22515; doi: 10.1038/srep22515 (2016).

## Supplementary Material

Supplementary Information

## Figures and Tables

**Table 1:** Values of criteria maximum concentrations (CMCs) recommended and 26 physical and chemical properties for seven transition metals.

**Properties**	**Abbreviation**	**Metals**							
		**Cd**	**Cr(III)**	**Cr(VI)**	**Cu**	**Hg**	**Ni**	**Ag**	**Zn**
Criteria maximum concentrations recommended	CMCs	2	570	16	13	1.4	470	3.2	120
Atomic number	*AN*	48	24	24	29	80	28	47	30
Relative atomic weight	*AW*	112.4	51.99	51.99	63.55	200.6	58.69	107.9	65.39
Atomic radius	*AR*	1.71	1.85	1.85	1.57	1.76	1.62	1.75	1.53
Covalent radius	*CR*	1.48	1.18	1.18	1.17	1.49	1.15	1.34	1.25
Pauling ionic radius	*r*	0.97	0.52	0.52	0.73	1.02	0.69	1.26	0.74
Melting point	*MP*	321	1857	1857	1085	−39	1453	961	420
Density of 300K	*D*	8.65	7.19	7.19	8.96	13.6	8.90	10.5	7.13
Heat of vaporization of the ionization potential change	*Eh*	99.57	344.3	344.3	300.3	59.23	370.4	250.6	115.3
Boiling point	*BP*	321	2672	2672	2567	357	2732	2163	907
Difference in ionization potentials between the ion oxidation numbers OX and OX^−1^	Δ*IP(eV)*	7.91	14.5	21.2	12.6	8.32	10.5	7.57	8.57
Electrochemical potential	Δ*E*_*0*_*(V)*	0.40	0.41	0.13	0.16	0.91	0.23	0.80	0.76
Electronegativity	*X_m_*	1.69	1.66	1.66	1.90	2.00	1.91	1.93	1.65
First hydrolysis constants	*|logKOH|*	10.1	4.00	4.00	8.00	3.40	9.90	12.4	8.20
Covalent index	*X*^*2*^_*m*_*r*	2.71	1.71	1.21	2.64	4.08	2.52	4.28	2.01
Polarization force parameters	*Z*^*2*^*/r*	4.21	14.5	81.8	5.48	3.92	5.80	0.87	5.41
Atomic ionization potential	*AN/*Δ*IP*	6.07	1.66	1.13	2.31	9.62	2.66	6.21	3.50
Softness index	*σp*	0.08	0.11	0.11	0.10	0.07	0.13	0.07	0.12
Ionization potential	*IP*	16.90	30.96	90.63	20.30	42.32	18.76	18.17	17.96
Electron density	*AR/AW*	0.02	0.04	0.04	0.02	0.01	0.03	0.02	0.02
Ionic charge	*Z*	2	3	6	2	2	2	1	2
Polarization force parameters	*Z/r*^*2*^	2.22	7.80	31.0	3.75	1.92	4.20	0.76	3.65
Similar polarization force parameters	*Z/AR*^2^	0.68	0.88	1.75	0.81	0.65	0.76	0.33	0.85
polarization force parameters	*Z/r*	2.11	4.84	13.6	2.74	1.96	2.90	0.87	2.70
Similar polarization force parameters	*Z/AR*	1.17	1.62	3.24	1.27	1.14	1.23	0.57	1.31
Electronegativity index	*x*	1.70	1.60	1.60	1.90	1.90	1.80	1.90	1.60
Relative softness (x is a electronegativity value index)	*Z/rx*	1.24	3.02	8.52	1.44	1.03	1.61	0.46	1.69

**Table 2:** Pearson product-moment parametric correlation of 26 characteristics of metal ions and the criteria maximum concentrations (CMCs) values by US EPA.

**Pearson’s product-moment correlation**			
**Ion characteristics**	**t**	***P***	**Correlation**
*σp*	4.47	0.004*	0.88
*CR*	−3.15	0.020*	−0.79
*AR/AW*	2.80	0.031*	0.75
*AN*	−2.68	0.037*	−0.74
*AW*	−2.64	0.039*	−0.73
*AN/*Δ*IP*	−2.59	0.041*	−0.73
*IR*	−2.55	0.043*	−0.72
*D*	−2.04	0.088*	−0.64
*X*^*2*^_*m*_*r*	−1.97	0.096*	−0.63
*MP*	1.94	0.100	0.62
*Eh*	1.87	0.111	0.61
*BP*	1.69	0.142	0.57
*x*	−1.44	0.200	−0.51
*X*_*m*_	−0.98	0.366	−0.37
Δ*E*_*0*_*(V)*	−0.94	0.385	−0.36
Δ*IP(eV)*	0.76	0.479	0.29
*Z/AR*^2^	0.56	0.596	0.22
*Z/AR*	0.46	0.664	0.18
*Z/rx*	0.42	0.693	0.17
*Z/r*	0.40	0.704	0.16
*AR*	−0.38	0.719	−0.15
*Z*	0.38	0.719	0.15
*Z/r*^*2*^	0.29	0.778	0.12
*|logKOH|*	−0.28	0.789	−0.11
*IP*	−0.27	0.799	−0.11
*Z*^*2*^*/r*	0.12	0.913	0.05

**Table 3:** Regression models with principal components for criteria maximum concentrations (CMCs) at natural logarithmic scale, where *R* ^2^ is the coefficient of determination, RSE is residual standard error, *P* is the statistical level of significance.

**Principal Components**	**Standard deviation**	**Proportion of Variance**	**Cumulative Proportion**	**Predictive Equations**	***R***^***2***^	**RSE**	***F***	***P***
*X_1_* = 0.567 *CR* + 0.568 *r* − 0.597	1.63	0.89	0.89	ln CMC = −8.75*X*_*1*_ + 13.34	**0.63**	1.58	10.23	0.019
*X_2_* = −0.586 *AW* − 0.596 *CR* − 0.549 *r*	1.60	0.86	0.86	ln CMC = −0.16*X*_*2*_ + 6.82	0.55	1.74	7.30	0.035
*X_3_* = 0.588 *AN* + 0.592 *CR* − 0.551 *r*	1.60	0.85	0.85	ln CMC = 0.059*X*_*3*_ + 6.23	0.54	1.76	7.03	0.038
*X_4_* = −0.46 *AN* + 0.362 *AW*−0.329 *CR* + 0.162 *r* + 0.723 *AR/AW*	2.06	0.88	0.88	ln CMC = −0.064*X*_*4*_ + 6.32	0.54	1.76	7.04	0.038

**Table 4:** Comparison among Criteria Maximum Concentrations (CMCs) predicted by the model based on integrated radius (IR), median lethal concentration (LC50) for the fresh water amphipod (*Hyalella azteca*, Crustacea) in Lake Ontario (Burlington city tap, Canada) in soft water (nominal) and soft water (measured), for group IIIB, group VB, group VIII, group IB and group IIB.

**Group**	**Period**	**Elements**	**Predicted CMC (μg/L)**	**(LC**_**50**_**) soft water (nominal) (μg/L)**	**(LC**_**50**_**) soft water (measured) (μg/L)**
IIIB	4	Sc	11	100	29
	5	Y	2.20	183	66
VB	4	V	89	989	1251
	5	Nb	27	250	26
	6	Ta	32	353	2
VIII	4	Co	61	16	16
	5	Rh	32	980	804
VIII	4	Ni	75	77	75
	6	Pt	42	131	110
IB	4	Cu	39	56	36
	5	Ag	1.64	1.72	0.25
	6	Au	0.92	841	446
IIB	4	Zn	35	70	56
	5	Cd	3.47	0.57	0.15

The data for LC_50_ values were collected by Borgmann *et al*.^40^.
