# Supplementary material for: Directly Predicting Water Quality Criteria from Physicochemical Properties of Transition Metals
Source: Sci Rep. 2016 Mar 3;6:22515. doi: 10.1038/srep22515 (PMC4776129; doi:10.1038/srep22515)
Supplement: Supplementary Information [file srep22515-s1.pdf]

The Authors: Ying Wang, Fengchang Wu, Yunsong Mu, Eddy Y. Zeng, Wei Meng, Xiaoli Zhao, John P. Giesy, Chenglian Feng, Peifang Wang, Haiqing Liao, and Cheng Chen

Manuscript entitled: *Directly Predicting Water Quality Criteria from Physicochemical Properties of Transition Metals*

Number Supplementary pages: 3

Number the Supplementary Figures: 3

Supplementary Figure Captions

Supplementary Figure S1. Models of USEPA-recommended criteria maximum concentrations (CMCs) plotted as natural logarithmic and seven physicochemical parameters of seven transition metal ions used in a of a single-parameter linear regression method, with natural logs of CMSs recommended by the USEPA labeled as ○. (A) Regression of natural logarithms of CMCs USEPA vs atomic number (*AN*) (R^2 = 0.544 and $P < 0.05$). (B) Regression of natural logarithms of CMCs USEPA vs atomic mass (*AM*) (R^2 = 0.536 and $P < 0.05$). (C) Regression of natural logarithms of CMCs USEPA vs relative covalent radius (*CR*) (R^2 = 0.623 and $P < 0.05$). (D) Regression of natural logarithms of CMCs USEPA vs Pauling ionic radius (*r*) (R^2 = 0.520 and $P < 0.05$). (E) Regression of natural logarithms of CMCs USEPA vs atomic ionization potential ($AN/\Delta IP$) (R^2 = 0.528 and $P < 0.05$). (F) Regression of natural logarithms of CMCs USEPA vs softness index (σp) (R^2 = 0.746 and $P < 0.05$). (G) Regression of natural logarithms of CMCs USEPA vs electron density (*AR/AW*) (R^2 = 0.567 and $P < 0.05$).

Supplementary Figure S2. Williams plot. The blue line represents residuals of ±3 standard deviations, while the red line represents the critical value of h*=0.75, training metals were labeled as ◊.

Supplementary Figure S3. Hat plot. The red line represents the critical value of h*=0.75. Hat values of training metals were labeled with ◊, and the hat values of predicted metals were labeled as ◊.

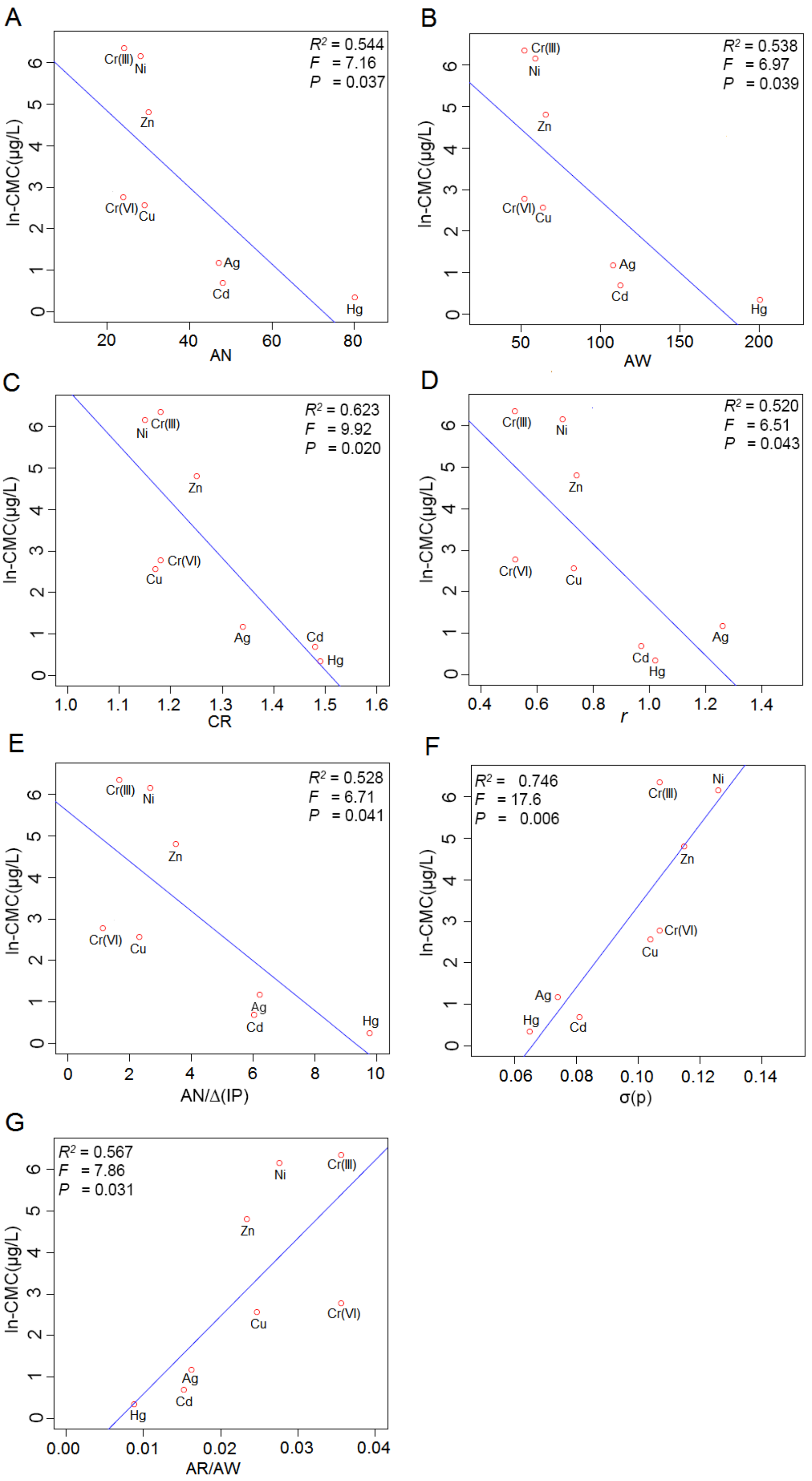
A
R^2 = 0.544
F = 7.16
P = 0.037
ln-CMC(μg/L)
AN
Cr(III)
Ni
Zn
Cr(VI)
Cu
Ag
Cd
Hg
B
R^2 = 0.538
F = 6.97
P = 0.039
ln-CMC(μg/L)
AW
C
R^2 = 0.623
F = 9.92
P = 0.020
ln-CMC(μg/L)
CR
D
R^2 = 0.520
F = 6.51
P = 0.043
ln-CMC(μg/L)
r
E
R^2 = 0.528
F = 6.71
P = 0.041
ln-CMC(μg/L)
AN/Δ(IP)
F
R^2 = 0.746
F = 17.6
P = 0.006
ln-CMC(μg/L)
σ(p)
G
R^2 = 0.567
F = 7.86
P = 0.031
ln-CMC(μg/L)
AR/AW

Figure S1

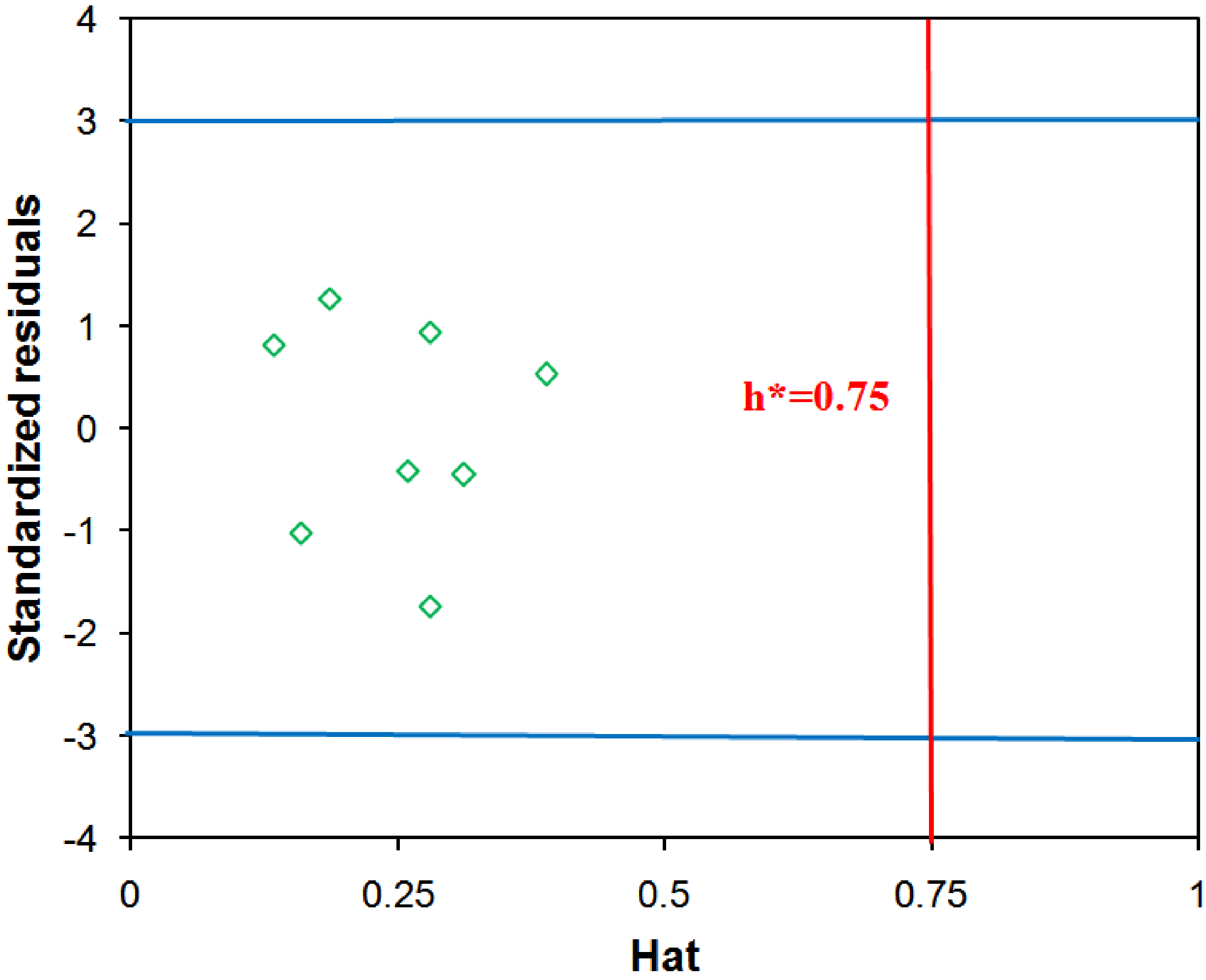

Figure S2

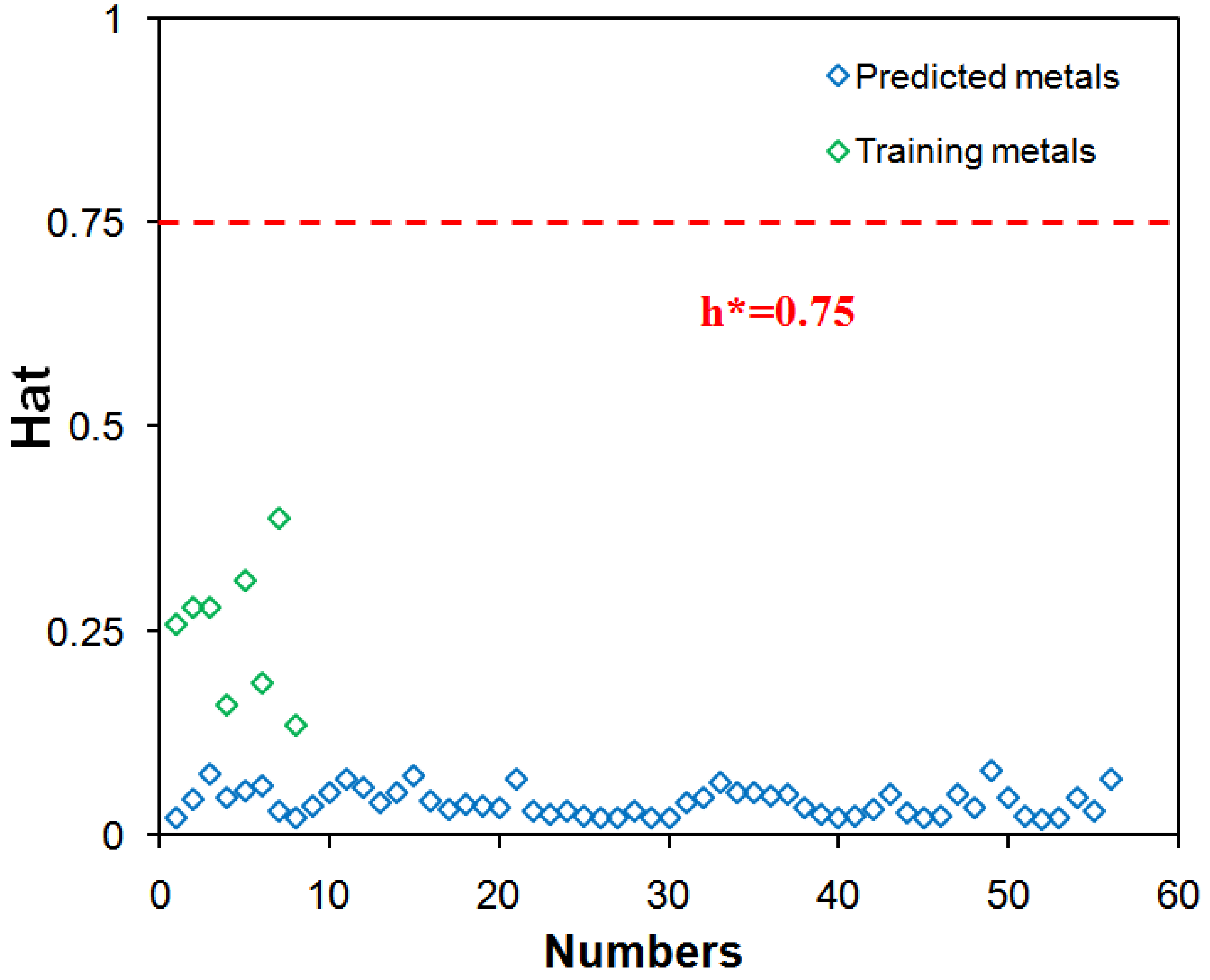
Predicted metals
Training metals
h*=0.75
Hat
Numbers
1
0.75
0.5
0.25
0
0
10
20
30
40
50
60

Figure S3
